# Neoadjuvant chemotherapy in breast cancer significantly reduces number of yielded lymph nodes by axillary dissection

**DOI:** 10.1186/1471-2407-14-4

**Published:** 2014-01-03

**Authors:** Thalia Erbes, Marzenna Orlowska-Volk, Axel zur Hausen, Gerta Rücker, Sebastian Mayer, Matthias Voigt, Juliane Farthmann, Severine Iborra, Marc Hirschfeld, Philipp T Meyer, Gerald Gitsch, Elmar Stickeler

**Affiliations:** 1 Department of Gynaecology and Obstetrics, University Medical Center Freiburg, Hugstetter Street 55, 79106 Freiburg, Germany; 2 Institute of Pathology, University Medical Center Freiburg, Hugstetter Street 55, 79106 Freiburg, Germany; 3 Department of Pathology Maastricht, Maastricht University Medical Center, PO Box 5800, 6202 AZ Maastricht, The Netherlands; 4 Institute of Medical Biometry and Medical Informatics, University Medical Center Freiburg, Stefan-Meier-Strasse 26, 79104 Freiburg, Germany; 5 Department of Nuclear Medicine, |University Medical Center Freiburg, Hugstetter Street 55, 79106 Freiburg, Germany; 6 Plastic and Aesthetical Surgery Freiburg, Bismarckallee17, 79098 Freiburg, Germany; 7 German Cancer Consortium (DKTK), Heidelberg, Germany; 8 German Cancer Research Center (DKFZ), Heidelberg, Germany

**Keywords:** Lymph node yield, Neoadjuvant chemotherapy, Lymphoid depletion, Breast cancer

## Abstract

**Background:**

Neoadjuvant chemotherapy (NC) is an established therapy in breast cancer, able to downstage positive axillary lymph nodes, but might hamper their detectibility. Even if clinical observations suggest lower lymph node yield (LNY) after NC, data are inconclusive and it is unclear whether NC dependent parameters influence detection rates by axillary lymph node dissection (ALND).

**Methods:**

We analyzed retrospectively the LNY in 182 patients with ALND after NC and 351 patients with primary ALND. Impact of surgery or pathological examination and specific histomorphological alterations were evaluated. Outcome analyses regarding recurrence rates, disease free (DFS) and overall survival (OS) were performed.

**Results:**

Axillary LNY was significantly lower in the NC in comparison to the primary surgery group (median 13 vs. 16; p < 0.0001). The likelihood of incomplete axillary staging was four times higher in the NC group (14.8% vs. 3.4%, p < 0.0001). Multivariate analyses excluded any influence by surgeon or pathologist. However, the chemotherapy dependent histological feature lymphoid depletion was an independent predictive factor for a lower LNY. Outcome analyses revealed no significant impact of the LNY on local and regional recurrence rates as well as DFS and OS, respectively.

**Conclusion:**

NC significantly reduces the LNY by ALND and has profound effects on the histomorphological appearance of lymph nodes. The current recommendations for a minimum removal of 10 lymph nodes by ALND are clearly compromised by the clinically already established concept of NC. The LNY of less than 10 by ALND after NC might not be indicative for an insufficient axillary staging.

## Background

Axillary lymph node status is one of the most powerful prognostic factors in breast cancer (BC) [1-3] and ALND the standard approach for local staging in lymph node positive patients. There is some evidence for an inverse correlation between a low number of removed axillary lymph nodes (often <10) and overall survival [4-10], which is controversially discussed [11-13].

Surgical staging of the axilla, particularly the number of positive lymph nodes is still a major driver for local and systemic treatment decisions. Therefore current guidelines recommend the removal of at least 10 lymph nodes [1,14], based on a mathematical model which determined the cut off at 10 lymph nodes to allow a 90% certainty of a true negative axillary status [14,15].

NC has become a common treatment for patients with locally advanced and lymph node positive BC. NC is able to downstage the number of involved axillary lymph nodes [16] as an important parameter in the definition of the pathological complete response (pCR). Clinical observations suggest a lower LNY after NC, which might be due to chemotherapy dependent parameters influencing detection rates. Therefore we examined these potential effects of NC by comparing retrospectively LNY by ALND in primary BC patients who underwent primary surgery versus NC and analysed its potential impact on clinical outcome.

## Methods

### Patients

We selected retrospectively 533 patients with primary BC from the database of the Department of Gynaecology and Obstetrics, University Medical Center Freiburg, who underwent ALND (Level 1 and 2) or sentinel node biopsy (SLNB) plus ALND from January 2001 to December 2010. Bilateral BC was excluded. All patients in the study had a histological proven axillary metastasis. The distinct algorithm for the confirmation of axillary involvement was a follows: In the case of a clinical suspicious axilla (91.8%, 167/182) the lymph nodes status was evaluated by core biopsies of distinct nodes before NC. With the proven lymph node metastasis patients underwent the consecutive NC. In the case of a clinical negative axilla, patients (8.2%, 15/182) underwent sentinel lymph node biopsy before NC.

Finally all patients (182(182) underwent the consecutive ALND after NC. The 182 patients receiving NC were defined as the primary chemotherapy group (PCG), who underwent consecutive surgery with standard ALND. Patients who received SLNB (15 patients, 8.2%) before NC and ALND after completion because of positive sentinel nodes were also included (total count including both sentinel and non-sentinel nodes, respectively). All patients received an anthracycline and/or taxane-based chemotherapy regime. Initial tumor size (Table 1) as well as response to NC was routinely measured by ultrasound. The primary surgery group (PSG; n = 351) was initially treated with primary surgery including standardized ALND, or SLNB (n = 193, 55.0%) and ALND because of positive sentinel node. Surgeries in both groups were performed by qualified breast surgeons according to a standardized protocol which is based on the national S3 guidelines and contains the comprehensive removal of the axillary tissue of the level 1 and 2 [17]. The influence of the individual surgeon on the LNY was evaluated.

**Table 1:** Patients and tumor characteristics

**Characteristic**	**PCG**		**PSG**		**P value**
	**N**	**%**	**N**	**%**	
N	182		351		
Mean age (range), y	49.82 (28–69)		60.33 (28–87)		< 0.0001
Mean tumor size, (range), mm	33.02 (0–100)		25.40 (2–89)		< 0.0001
Histology					0.219
Invasive ductal	151	82.9	270	76.9	
Invasive lobular	21	11.5	60	17.0	
Others	10	5.5	21	5.9	
Tumor stage					<0.0001
T1	22	12.0	156	44.4	
T2	116	63.7	157	44.7	
T3	19	10.4	26	7.4	
T4	25	13.7	12	3.4	
Nodal status					<0.0001
pN0	82	45.1	54	15.4	
pN1	63	34.6	189	53.8	
pN2	37	20.3	108	30.8	
Grading					0.0062
G1	6	3.3		9.9	
G2	119	65.4	219	62.4	
G3	57	31.3	97	27.6	
Lymphovascular					<0.0001
invasion					
L0	131	71.8	168	47.9	
L1	51	28.0	183	52.1	
Hormone receptor					0.004
status					
ER positive	131	71.9	261	74.3	
PR positive	96	52.7	225	64.1	
Menopausal status					<0.0001
Premenopausal	78	42.6	92	26.2	
Postmenopausal	104	57.1	259	73.8	
Mastectomy					0.010
Yes	76	42.3	189	53.8	
No	106	57.7	162	46.2	
HER2neu status					0.003
Positive	47	25.8	53	15.0	

Patients and tumor characteristics including age, size, histological subtype, stage, nodal status, grading, lymphovascular invasion, estrogen, progesterone and Her2 receptor status, menopausal status and type of surgery were evaluated (Table 1). The tissue specimens were analyzed by 19 pathologists and the potential impact of the individual investigator on LNY separately analyzed. All lymph nodes were processed and analyzed by a standardized protocol according to the current national S3 guidelines, and inconclusive cases were subjected to immunohistochemistry (IHC) [17]. Sentinel nodes were serially sectioned, and submitted to hematoxylin-eosin as well as IHC against pancytokeratin.

A consecutive series of 94 lymph nodes from PCG and 97 from PSG, respectively, were re-evaluated for differences regarding the following histological features: median size, capsular invasion, diffuse fibrosis, lymphoid depletion, B-and T-cell accentuated depletion, signs of bleeding and calcification.

Outcome analyses regarding local, regional and distant recurrences as well as 5 year DFS and OS were performed for all subgroups of patients in regards to LNY, axillary response and histomorphological features, respectively. These data consider the consecutive treatment in both groups, which presented equally balanced with comparable endocrine as well as radiation therapy rates (data not shown).

### Statistical analyses

Lymph node counts were described by medians and quartiles and compared between groups using Wilcoxon’s test. For univariate comparisons between groups, Welch’s t-test (for continuous variables) and Pearson’s Chi square test (for nominal variables) were used. For descriptive aims, box plots were produced. Further, lymph node counts were analyzed in a multivariable Poisson regression analysis to adjust for a number of pre-specified covariates. Time-to-event endpoints were analyzed using Cox’s regressions model, and five-year event free rates with confidence intervals were taken from the regression-based survival functions. The significance level was set to alpha = 0.05. Data analysis was performed by open statistical software environment R (R Development Core Team, “R: A Language and Environment for Statistical Computing”. R foundation for Statistical Computing, 2009. URL http://www.R-project.org).

## Results

We included 182 patients receiving NC, and 351 patients receiving primary surgery into this retrospective analysis. Significant differences were seen in a variety of baseline criteria between the two groups, since treatment decisions in regards to NC are routinely based on tumor size, clinical nodal involvement, grading, age and receptor status, though no significant difference in the histological tumor type was seen (Table 1).

**Figure 1:**
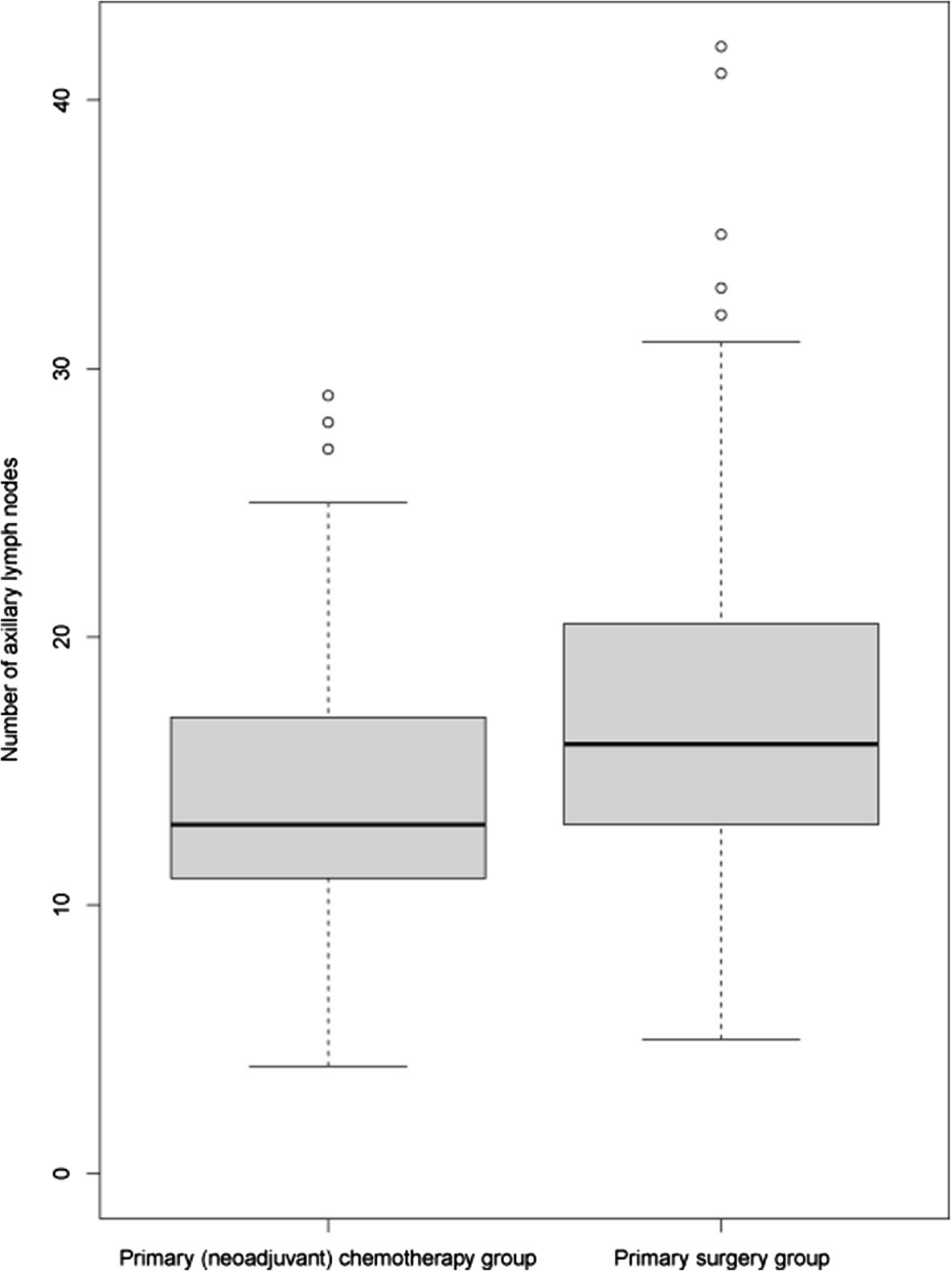
**Statistical analysis: total number of yielded axillary lymph nodes.** The total number of detected nodes is shown by box plots for the patients treated with neoadjuvant chemotherapy and primary surgery (P = 0.001, Wilcoxon’s test). Thick lines, median (50% percentile); gray boxes, 25% to 75% percentile; thin lines, minimal and maximal value.

Our analyses revealed a significantly lower LNY in patients undergoing NC in comparison to patients who did not. With a median total number of 13 nodes (interquartile range 11–17) in the PCG compared to 16 axillary nodes in the PSG (interquartile range 13–20), these results were highly statistically significant (p < 0.0001; Figure 1). Since the removal of at least 10 axillary nodes represents the gold standard for systematic axillary staging, we dichotomized the number of removed lymph nodes using 10 nodes as the cut-off. The analysis found a significantly higher number of patients (27 pts., 14.8%) in the PCG with less than 10 yielded lymph nodes in comparison to 12 patients (3.4%) in the PSG (p < 0.0001).

As expected the number of patients with involved lymph nodes was significantly lower in PCG (100/182, 54.9%) compared to the PSG (297/351, 84.6%; p < 0.0001). For the PCG, status of lymph node positivity after NC at the time of surgery did not influence the number of retrieved lymph nodes. We found a median total number of 13 lymph nodes (interquartile range 10–17) for patients with nodal involvement compared to 14 nodes (interquartile range 11–18) in patients without nodal involvement (p = 0.654), respectively, (Table 2).

**Table 2:** Influence of nodal involvement on total lymph node yield

	**Median total number of lymph nodes for patients with nodal involvement**	**Median total number of lymph nodes for patients without nodal involvement**	**P-value**
PCG	13.0 (10–17)*	14.0 (11–18)*	0.654
	PCG	PSG	
Median number of involved lymph nodes	3.0 (1–6)*	2.5 (1–5)*	0.904

* interquartile range.

Furthermore the median number of involved nodes/total nodes was comparable for both groups with a median number of 3.0 positive nodes/13 total number in the PCG and a median number of 2.5 involved nodes/17 total number of nodes in the PSG (p = 0.904), respectively (Table 2).

After adjusting for age, menopausal status, histological subtype, tumor size, grading, lymphovascular invasion, receptor status, HER2 status, mastectomy and NC, the multivariate Poisson regression analysis confirmed neoadjuvant treatment as the strongest independent factor for a lower LNY after ALND (p < 0.0001). In addition, age represented an independent factor with decreased LNY with increasing age (p = 0.031; Table 3).

**Table 3:** Influence of clinico-pathological characteristics total lymph node yield in 533 breast cancer patients

**Variable**	**Relative risk**	**95% CI**		**P-value**
Intercept	17.78123	15.26726	20.70916	0.00000
Group				
Primary chemotherapy	0.83132	0.78241	0.88328	0.00000
Primary surgery	1.0*			
Age (per year)	0.99735	0.99560	0.99911	0.00313
Mean tumor size	1.00051	0.99825	1.00277	0.65826
Histology				
Invasive lobular	0.98133	0.91887	1.04803	0.57423
Others	0.99680	0.90075	1.10309	0.95052
Tumor stage				
T2	0.96670	0.90878	1.02832	0.28274
T3	0.98958	0.86080	1.13762	0.88293
T4	0.86859	0.76246	0.98949	0.03410
T1	1.0			
Nodal status				
N1	0.99459	0.93456	1.05848	0.86435
N2	1.03164	0.95485	1.11461	0.42991
N0	1.0			
Grading				
G2	1.07546	0.98631	1.17266	0.09941
G3	1.10697	1.00705	1.21680	0.03525
G1	1.0			
Hormone receptor status				
ER positive	0.98314	0.91915	1.05160	0.62056
ER negative	1.0			
PR positive	1.06930	1.00638	1.13616	0.03035
PR negative	1.0			
HER2neu status				
Positive	1.05745	0.99583	1.12288	0.06822
Negative	1.0			
Type of breast surgery				
Mastectomy	0.97233	0.92656	1.02036	0.25405
Breast conserving surgery	1.0			

*reference value.

Since NC is employed for downstaging of local disease, the LNY by ALND might be compromised by the regression grade as treatment response. Regression was classified by a semiquantitative scoring system according to Sinn [16], ranging from 0 to 4 (0 = no effect, 1 = resorption and tumorsclerosis, 2 = minimal residual invasive tumor < 0.5 cm, 3 = residual non-invasive tumor only, 4 = no tumor detectable). However, no correlation between the regression grade and LNY was found (data not shown). An isolated view on the 28.6% (n = 52) patients with an axillary pathological complete response (pCR) revealed no influence of response on LNY (p = 0.615). Furthermore the regional axillary recurrence rates were statistically not different between these patients (1.9%) and those (3.0%) who did not achieve an axillary pCR.

To evaluate the potential impact of the individual surgery or pathological examination we also investigated the dependence of LNY on the individual surgeon or pathologist, respectively. Fifteen surgeons, with six performing 75.8% of the ALNDs (404/533) and 19 pathologists, with five main specialists, performing 81.2% of the examinations (449/533), were involved. Univariate and multivariate analyses confirmed the independence of the LNY from individual surgeon pathologist as well as their specific interactions (data not shown).

Since NC is known to alter histomorphology of lymph nodes [18], including size and depletion of lymphocytes, which could potentially compromise their detectability, we investigated these features in more detail. In a consecutive series of 191 lymph node specimens, from 94 patients of PCG and 97 patients of PSG the histomorphological parameter median size, capsular invasion, diffuse fibrosis, lymphoid depletion, B-and T-cell accentuated depletion, signs of bleeding and calcification, were evaluated respectively.

*Diffuse fibrosis* within a lymph node was defined and scored as 0 = no presence, 1 = partial presence, 2 = clear presence, 3 = strong presence of collagen fibers. *Lymphoid depletion* was classified by the density of lymphocytes and scored as 0 = no reduction, 1 = reduction up to 30%, 2 = reduction up to 50%, 3 = reduction up to 90%, respectively. The depletion was separately evaluated for a *B-cell* accentuated depletion with a decrease in the B-cell zone or a *T-cell* accentuated in the T-cell zone (interfollicular). *Signs of bleeding* was defined as the appearance of macrophages phagocytazing hemosiderin, or cholesterol crystals, respectively, and scored as 0 = no macrophages, 1 = single macrophages, 2 = up to 50%, 3 = up to 100% of the area. The presence of calcification was defined as a sign of older necrosis. All findings and their statistical analyses are summarized in Table 4.

**Table 4:** Histomorphological criteria of lymph nodes

**Characteristic**	**PCG**	**PSG**	**P value**
	**N**	**N**	
N	94	97	
Median lymph node size	8.71	8.39	0.6289
Capsular invasion			0.0007
Yes	26	51	
No	68	46	
Diffuse fibrosis			<0.0001
0	31	78	
1	22	17	
2	19	2	
3	22	0	
Lymphoid depletion			<0.0001
0	19	86	
1	54	11	
2	19	0	
3	2	0	
B-cell accentuated			<0.0001
0	30	91	
1	64	6	
T-cell accentuated			<0.0001
0	64	93	
1	30	4	
Signs of bleeding			<0.0001
0	77	96	
1	15	1	
2	1	0	
3	1	0	
Calcification			0.0002
Yes	81	97	
No	13	0	

NC had a profound effect on the histomorphological appearance of lymph nodes. The features diffuse fibrosis, lymphoid depletion and signs of bleeding were more frequent in the PCG, while the capsular invasion and lymphangiosis carcinomatosa due to the supposed treatment effects were less frequent in comparison to the PSG. However, the multivariate analysis identified solely the parameter lymphoid depletion as an independent predictive factor for a lower LNY (Table 5), but not as a predictor for a complete axillary response (p = 0.662).

**Table 5:** Influence of histomorphological features on total lymph node yield in a subgroup of consecutive 191 breast cancer cases

**Variable**	**Relative risk**	**95% CI**		**P-value**
Intercept	15.81943	14.28861	17.51425	0.00000
Group				
Primary chemotherapy	0.94772	0.84264	1.06592	0.37055
Primary surgery	1.0*			
Median lymph node size	0.99747	0.98930	1.00574	0.54908
Capsular invasion	0.90984	0.82697	1.00103	0.05251
Diffuse fibrosis				
1	1.02911	0.91502	1.15743	0.63216
2	0.97344	0.81156	1.16761	0.77174
3	0.84740	0.69909	1.02717	0.09163
0	1.0			
Lymphoid depletion				
1	0.72503	0.59371	0.88540	0.00161
2	0.69376	0.52246	0.92123	0.01150
3	0.54756	0.33636	0.89139	0.01542
0	1.0			
B-cell accentuated				
1	1.11951	0.92850	1.34982	0.23691
0	1.0			
T-cell accentuated				
1	1.22733	1.06029	1.42068	0.00606
0	1.0			
Signs of bleeding				
1	0.89257	0.66532	1.19746	0.44844
2	1.92896	0.75390	4.93552	0.17049
3	0.81796	0.37759	1.77194	0.61041
0	1.0			
Calcification				
1	1.25871	0.92286	1.71677	0.14622
0	1.0			

*reference value.

To evaluate the clinical impact of these findings we performed additional outcome analyses. The follow up rates were 92.3% in the PCG and 91.5% in the PSG, respectively. Local and regional recurrences were nearly identical (Table 6) with a five-year local recurrence free survival rate of 95.0% in the PCG and 94.8% in the PSG (p = 0.944), respectively. However we detected significant differences for distant recurrences with a five-year distant metastasis free survival of 80.6% in the PCG and 91.5% in the PSG (p = 0.00113), respectively.

**Table 6:** Clinical outcome depending on primary treatment

	**PCG (n = 168)**	**PSG (n = 321)**	**Hazard ratio (HR) with 95% CI**			**P-value**
	**n**	**n**				
Local recurrence	8	14	0.9694	0.4066	2.3111	0.944
Regional recurrence	5	0	0.0000	0.0000	infinity	0.999
Distant recurrence	32	25	0.4194	0.2485	0.7077	0.001
End of disease-free survival	39	61	0.8453	0.5655	1.2633	0.412
Death for all reasons	32	51	0.8729	0.561	1.3581	0.547
Five-year local recurrence free survival rate	95.0%	94.8%				
95% confidence interval (CI)	[91.7%; 98.4%]	[92.1%; 97.7%]				
Five-year distant metastasis free survival rate	80.6%	91.5%				
95% confidence interval (CI)	[74.6%; 87.2%]	[88.3%; 94.8%]				
Five-year disease free survival rate	77.0%	80.1%				
95% confidence interval (CI)	[70.7%; 83.8%]	[75.7%; 84.9%]				
Five-year overall survival rate	82.0%	84.3%				
95% confidence interval (CI)	[76.2%; 88.2%]	[80.3%; 88.6%]				

The five-year DFS(OS) was 77.0% (82%) for the PCG and 80.1% (84.3%) (p = 0.412; (p = 0.547)) for the PSG, respectively, (Table 6).

The subgroup analyses for the PCG with an incomplete staged axilla (less than 10 nodes) did also not detect significant differences for clinical outcome in regards to local, regional and distant recurrences, respectively.

## Discussion

It is well known that NC could result in downstaging of positive axillary lymph nodes [16] but the potential influence of chemotherapy on the LNY and their morphology and detectibility is still unclear. Since an incomplete axillary staging might compromise further treatment modalities (e.g. radiation therapy) and curation rates, we examined over 500 patients in regards to this pertinent clinical question. We were able to identify clearly NC as a significant and independent factor for a reduced LNY (13 in the PCG vs. 16 in the PSG; p < 0.0001) by ALND. Our findings are in line with several smaller recently published studies which found similar decreased numbers [19-21]. The outcome analyses clearly indicated, that a reduced LNY did not affect five-year DFS as well as OS.

The rate of suboptimal staged axillae with less than 10 lymph nodes was also significantly higher in the PCG (14.8% vs. 3.4%; p < 0.0001). This phenomenon was also seen in two other trials with rates ranging from 13% [21] to 45% [20], respectively. As an important aspect, we could clearly exclude a negative effect on local, regional and distant recurrence rates, as well as DFS and OS, respectively.

Our two groups were significantly heterogeneous regarding a variety of baseline criteria, esp. tumor size and clinical nodal involvement, which both were markedly advanced in the PCG. This might be clinically reflected by the fact, that the rate of distant metastases is significantly higher in this group. The missing impact on OS might be due to the relatively limited follow up. However, applying multivariate analyses, we could exclude a significant influence of the described heterogeneity on our findings. Noteworthy, the known effect of an increasing age lowering the LNY [22], which was also significant in our cohort, did not lead to a lower LNY in our PSG, which was significantly older with a mean age of 60.3 years versus 49.8 years for the PCG. These results support our hypothesis of NC as the strongest variable for a diminished lymph node number.

The expertise of the individual surgeon as well as pathologist on the LNY was considered a strong predictor for the LNY [20,22,23]. However, in our study cohort multivariate analyses could not detect any significant influence of lymph node retrieval by the involved specialists as well as specific interactions between them. These findings strongly support the suggested profound impact of the NC itself on the lymph node detection frequency. The biological effect of chemotherapy is clearly visible by the detected downstaging effect with a lower number of lymph node positive patients (54.9% vs. 84.6%; p < 0.0001) in the PCG. These findings are in line with the NSABP-B18 trial, which found a comparable effect with lymph node metastasis in 41% neoadjuvant treatment group compared to 57% in the postoperative chemotherapy group [24]. Nevertheless, as a consistent overall finding, axillary pCR did not alter the LNY in our study or influenced the regional recurrences rates.

Still, it remains unclear, if the observed biological effects of NC affect the histopathological work up and cause the observed lower detection rates. We revealed significant chemotherapy induced histomorphological changes within lymph nodes regarding the features lymphoid depletion, diffuse fibrosis, calcifications and signs of bleeding. Multivariate analyses identified lymphoid depletion as an independent histomorphological parameter for a lower LNY after NC. This might be explained by the fact that lymphoid depletion will lead to shrinkage of lymph nodes as well as to regression of lymphoid tissue. Both effects could hamper their detectability. These findings are in line with recently published studies which reported also chemotherapy-induced changes in lymph nodes including lymphoid depletion [18,25,26]. These signs were suggested to be surrogates for previous lymph node metastasis which responded completely to therapy [27]. Clinically, this is of high importance, since a broad number of studies have already correlated clinical and pathologic primary tumor responses with outcome [24,28-32]. However in our study, lymphoid depletion was not associated with a higher axillary response rate (pCR).

Since treatment response might be also an additional potential factor affecting the LNY, our present study evaluated also the pathological tumor response according to Sinn by a standardized classification system for the regression grade [16]. Interestingly, the histopathologically evaluated response rates had no significant impact on LNY, specifically the axillary response rates did not influence the LNY. Furthermore, axillary response was no predictor for later axillary recurrence.

## Conclusion

In conclusion, our study on more than 500 patients with primary BC clearly identified NC as a significant independent parameter for a reduced LNY by ALND. Furthermore, the NC concept had profound effects on the histomorphological appearance of lymph nodes. Lymphoid depletion was a strong independent factor for a lower number of yielded axillary lymph nodes after NC. These histological changes could hamper the detectability of lymph nodes which was investigator independent. However, the lower LNY had no impact on clinical outcome. The still existing recommendations for a minimum removal of 10 lymph nodes by ALND are clearly compromised by the clinically already established concept of NC. Consequently, the lymph node count of less than 10 by ALND after NC is not indicative for an insufficient axillary staging. Therefore, guideline recommendations for the future should consider the combination of both innovative treatment modalities.

### Ethical standards

The institutional ethical review board of University of Freiburg, review board approved the investigation protocol with the number 324/09.

### Patient consent

Written informed consent was obtained from all patients for the publication of this report and any accompanying images.

## Competing interest

None of the authors has to declare any competing financial or non-financial conflict of interest in regard to this work.

## Authors’ contributions

TE, ES, PTM and GG compiled study design and contributed equal effort to this work. TE, ES and PTM wrote the manuscript. Data acquisition, analysis and interpretation was performed by, ES, GG, GR, PTM, MOV and AzH. Final approval of data analysis and manuscript were conducted by SM, JF, SI, MH and MV. All authors read and approved the final manuscript.

## Pre-publication history

The pre-publication history for this paper can be accessed here:

http://www.biomedcentral.com/1471-2407/14/4/prepub
